# Methicillin-susceptible, Doxycycline-resistant *Staphylococcus aureus*, Côte d’Ivoire

**DOI:** 10.3201/eid1303.060729

**Published:** 2007-03

**Authors:** Olivier Lesens, Rachel Haus-Cheymol, Philippe Dubrous, Catherine Verret, André Spiegel, Richard Bonnet, Michèle Bes, Henri Laurichesse, Jean Beytout, Jerome Etienne, René Migliani, Jean Louis Koeck, the Working Group on Cutaneous Infections in the Army

**Affiliations:** * Centre Hospitalier Universitaire, Clermont-Ferrand, France; † Ecole du Val de Grâce, Paris, France; ‡ Hôpital d'Instruction des Armées Robert-Picque, Bordeaux, France; § Université Lyon 1, Lyon, France; ¶ Institut de Médecine Tropicale du Service de Santé des Armées, Le Pharo, Marseille-Armées, France

**Keywords:** Abscess, carrier state, community-acquired infections, leukocidins, nasal carriage, soft tissue infections, staphylococcal skin infections, Staphylococcus aureus, dispatch

## Abstract

We report 2 outbreaks of Panton-Valentine leukocidin–positive, doxycycline-resistant, methicillin-susceptible *Staphylococcus aureus* infections in French soldiers operating in Côte d’Ivoire. In a transssectional survey, nasal carriage of this strain was found in 2.9% of 273 soldiers about to be sent to Côte d’Ivoire and was associated with prior malaria prophylaxis with doxycycline.

## Abstract 2

This virulent clone has already spread to other continents.

*Staphylococcus aureus* strains that produce Panton-Valentine leukocidin (PVL) have been shown to be associated with community-acquired infections such as skin and soft tissue infections and necrotizing pneumonia (*1*). A limited number of PVL-positive, methicillin-resistant *S. aureus* (MRSA) clones have recently emerged globally (*2*) and have been described as causing epidemic community-acquired infections (*3*,*4*). In contrast, PVL-positive, methicillin-susceptible *S. aureus* (MSSA) corresponds to many diverse clones (*5*) that may cause sporadic infections with limited dissemination. We describe severe staphylococcal infections due to a clone of doxycycline-resistant, PVL-positive (doxyR-PVL+) MSSA in soldiers who served in Côte d’Ivoire.

## The Study

During 2004 and 2005, 4 soldiers with recurrent cutaneous infections related to doxycycline-resistant MSSA (DoxyR-MSSA) visited the Centre Hospitalier Universitaire in Clermont-Ferrand, France. The soldiers belonged to 2 companies, A and B, based at different places in France (Clermont-Ferrand and Brives, respectively), and had been to Côte d’Ivoire at 2 distinct periods. In October 2005, a health warning was sent to French military authorities. We conducted retrospective interviews of soldiers in company A and performed a transsectional nasal carriage survey of 273 soldiers in company B, who were about to be sent to Côte d’Ivoire. We defined case-patients as soldiers who had had at least 1 cutaneous infection during or after their time in Côte d’Ivoire*.* Information was collected from company A case-patients by telephone interview and, for hospitalized soldiers, chart review.

When available, staphylococcal strains were sent to the French National Reference Centre for Staphylococci in Lyon, France. Sequences specific for staphylococcal enterotoxin genes (*sea-e, seh*, *sek*, *sem*, *sel,* and *seo*), the PVL genes (*luk*S*-*PV*-luk*F*-*PV), and accessory gene regulator alleles (*agr* 1–4) were detected by PCR (*6**–**8*). Fingerprinting by pulsed-field gel electrophoresis, *spa* typing, and multilocus sequence typing were performed as described (*9*).

Company A comprised 70 French soldiers who had taken doxycycline, 100 mg per day, for malaria prophylaxis while in Côte d’Ivoire (August–November 2003). During their 3 weeks in training camp, each soldier stayed in 1 of 4 rooms. Of 13 soldiers who stayed in room 3, 8 (61.5%) reported having had at least 1 cutaneous infection while in Côte d’Ivoire, compared with none of 2 soldiers in room 1, 1 (8%) of 13 in room 2, and 7 (18%) of 39 in room 4. Two soldiers (1A and 2A) visited our clinic in November 2004 for treatment of abscesses that required surgical debridement for 1 year. For each patient, doxyR-MSSA was isolated from the abscess in a site acting as a reservoir (nasal or perianal skin). Their conditions were successfully treated with topical application of mupirocin to the reservoir site. No recurrence occurred after a year. Another soldier from company A (patient 3A) was found to be an asymptomatic nasal carrier of doxyR-MSSA. His wife (patient 4A) experienced several recurrent doxyR-MSSA abscesses, including 1 that was debrided at our hospital in January 2004.

In company B, ≈70 soldiers had served in Côte d’Ivoire during October 2004–February 2005 and stayed for 6 weeks in the same training camp that company A stayed in the year before. Two soldiers (1B and 2B) visited our hospital. Patient 1B was hospitalized with furunculosis and osteomyelitis of the left femoral diaphysis 5 months after attending training camp (and 1 month after he returned to France). Surgically removed bone samples contained *S. aureus* isolates that had profile characteristics similar to those obtained from furuncles (Figure). Patient 2B visited our clinic in March 2005 for recurrent axillary abscesses due to the same clone of MSSA. He did not experience recurrence after MSSA eradication from the nasal and axillary reservoirs. In September 2005, recurrent furunculosis developed in a third soldier (3B), who had not gone to Côte d’Ivoire but who was in close contact with soldiers returning from there. In April 2005 and December 2005, doxyR-MSSA–related recurrent abscesses developed in his wife and their 5-year-old child.

**Figure:**
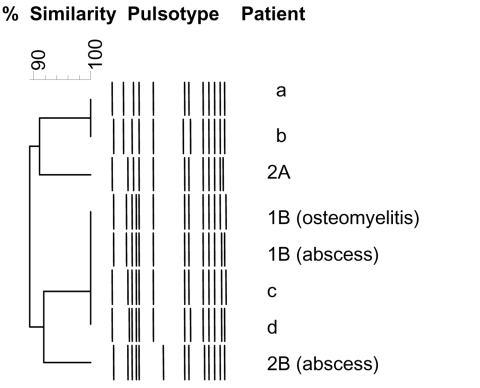
Dendogram constructed from the schematic representation of the pulsed-field gel electrophoresis types of 4 epidemic methicillin-susceptible *Staphylococcus aureus* (MSSA) isolates included in this study (patients 2A, 1B, and 2B); 1 strain subsequently isolated from an abscess in a soldier belonging to company A, who had been in Côte d’Ivoire in October 2005 (patient b); and 3 MSSA strains isolated from abscesses in soldiers belonging to a company other than A or B (patients a, c, d). Isolates from all patients had the following genetic characteristics: *agr* type 3, positive for Panton-Valentine leukocidin, negative for *mec*A gene, and toxin genes *sea, seh, and sek.* In addition, isolates from patients a and 1B were sequence type 1 and *spa* type 590.

To estimate the prevalence of PVL-positive MSSA carriage in soldiers, nasal culture specimens were collected on May 9 and 10, 2005, from 273 soldiers in company B (a total of 1,100 soldiers) who were about to be sent to Côte d’Ivoire. Of these 273 soldiers, 98 (35.9%) were colonized with *S. aureus.* Among them, 28 (10%) were carriers of PVL–doxyR-MSSA and 8 (2.9%) were carriers of PVL+doxyR-MSSA. These PVL+doxyR-MSSA isolates belonged to the same clone as those found in patients 2A, 1B, and 2B. All the MSSA isolates were considered to belong to the same clone as they shared a large core of common genetic characteristics (Figure). All PVL+doxyR-MSSA carriers had been to Côte d’Ivoire and had taken doxycycline for malaria prophylaxis (Table).

**Table:** Factors associated with nasal carriage of *Staphylococcus aureus* in 273 soldiers (company B) who were about to be sent to Côte d’Ivoire

Variable	PVL+DoxyR *S. aureus*† (n = 8)	PVL–DoxyR *S. aureus*‡ (n = 34)	DoxyS *S. aureus*§ (n = 56)	p value¶ (PVL+ vs. PVL–)	p value¶ (PVL+DoxyR vs. PVL–DoxyR)
Age, y	24.3	25.9	24.5	NS	NS
Male (%)	87.5	97.1	100.0	NS	NS
Living with health care worker (%)	25.0	26.5	12.5	NS	NS
Living with children <16 y (%)	50.0	61.8	53.6	NS	NS
Hospitalization within 1 year (%)	0.0	17.6	17.8	NS	NS
Fight sport practice# (%)	0.0	5.9	7.1	NS	NS
Previous mission in malaria-endemic area (%)	100.0	91.2	37.5	<0.001	NS
Previous mission in Côte d’Ivoire (%)	100.0	88.2	30.4	<0.001	NS
Previous doxycycline intake (%)	100.0	88.2	32.1	<0.001	NS

*PVL, Panton-Valentine leukocidin; DoxyR, doxycycline resistant; DoxyS, doxycycline susceptible; NS, not significant. †All methicillin-susceptible *S. aureus* (MSSA). ‡28 MSSA and 6 methicillin-resistant *S. aureus* (MRSA). §55 MSSA and 1 MRSA, all PVL-negative. ¶Fisher exact test. #Military exercises that involve physical contact.

## Conclusions

We characterized 2 outbreaks caused by the same clone of PVL+doxyR-MSSA. These outbreaks occurred in 2 military companies that served in Côte d’Ivoire at different times and whose soldiers received doxycycline for malaria prophylaxis. This epidemic MSSA clone was responsible for infections traditionally associated with PVL, mainly skin and soft tissue infections, but also deep-seated infections such as severe osteomyelitis. Since these outbreaks, several similar cases affecting different companies who had been to Côte d’Ivoire have been reported to the French military authorities (Figure). Another striking feature was the spread of the clone within families after the soldiers’ return from Côte d’Ivoire. This virulent clone has already disseminated to different continents. Analysis of the database of the French National Reference Centre for Staphylococci (which contains the characteristics of ≈5,000 *S. aureus* strains worldwide) found 25 methicillin-susceptible isolates with identical toxin gene content and *agr* type from unrelated patients from Africa, Polynesia, and France (data not shown).

Our study has limitations. The investigation conducted in company A was retrospective, so we could not control for other pathogens. Recall bias may have occurred, and the incidence of cutaneous infections in company A could have been underestimated. However, epidemiologic links between case-patients were well established. Only case-patients who visited our hospital were documented, but they enabled recognition of the outbreaks. Recent similar cases in soldiers who visited our hospital for PVL+doxyR-MSSA cutaneous infections after their return from Côte d’Ivoire confirm that the strain is still disseminating and is strongly associated with a stay in that country (Figure).

The infecting MSSA is remarkable in its resistance to doxycycline, which may favor selection of a preexisting PVL+doxyR-MSSA in carrier soldiers who are given a prophylactic dose of doxycycline. Because the 2 companies had no contact with each other before, during, and after their stay in Côte d’Ivoire, we think that the *S. aureus* clone persists in Côte d’Ivoire. Transmission may have occurred through the persistence of the strain in the environment of the training camp in Côte d’Ivoire, where hygiene conditions were poor (4 rooms, 3 showers, 3 lavatories for ≈70 soldiers). However, the relatively high prevalence of PVL-positive MSSA carriage found in our study suggests that nasal carriage may play an important role in transmission of these highly virulent microorganisms.
